# Editorial: Do We Need Socio-Emotional Skills?

**DOI:** 10.3389/fpsyg.2021.723470

**Published:** 2021-08-06

**Authors:** Daniel Danner, Clemens M. Lechner, Marion Spengler

**Affiliations:** ^1^University of Applied Labour Studies, Mannheim, Germany; ^2^GESIS Leibniz Institute for the Social Sciences, Mannheim, Germany; ^3^Medical School Berlin, Berlin, Germany

**Keywords:** socio-emotional skills, personality, non-cognitive skills, character strengths, life outcomes

The question we chose for this Research Topic—Do we need socio-emotional skills?—is deliberately broad. It can be asked on different levels: Do we as individuals need socio-emotional skills to achieve success, health, and happiness? Do we as researchers studying individual differences need data on socio-emotional skills to unravel the determinants of life success over and above cognitive abilities? Do we as organizations need to select applicants with socio-emotional skills, as they will show better performance in the future? Finally, do we as a society need socio-emotional skills to understand or overcome social inequalities?

The articles in this Research Topic offer promising new insights that support the view that socio-emotional skills can be useful on each of these levels. These articles contribute to three strands of the literature on socio-emotional skills: the conceptualization and definition of socio-emotional skills, the relevance of socio-emotional skills for success at school and at work, and how best to foster socio-emotional skills.

## Definition and Conceptualization: What are Socio-Emotional Skills?

*Socio-emotional skills* is an umbrella term used to describe psychological constructs such as personality traits, motivation, or values (e.g., Duckworth and Yeager, 2015; Lechner et al., 2019). Closely related terms are “character strengths,” “non-cognitive skills,” “soft skills,” and “twenty-first-century skills” (e.g., De Fruyt et al., 2015; Abrahams et al., 2019). The common denominator is that these terms describe functional capacities that allow individuals to work efficiently and persistently, build trusting relationships with others, cope with stress and setbacks, lead and motivate others, and be creative and explore novel ideas.

It is readily apparent that socio-emotional skills have a lot in common with the Big Five personality traits, Conscientiousness, Agreeableness, Emotional Stability (Negative Emotionality), Extraversion, and Open-Mindedness. Indeed, the Big Five model is currently the most widely used framework to assess socio-emotional skills. Although the distinction between socio-emotional skills, personality traits, and related constructs is sometimes blurred, there are subtle differences (e.g., Soto et al., 2021): whereas personality traits describe characteristic patterns of feelings, thoughts, and action (i.e., typical behaviors), socio-emotional skills describe how well individuals can perform specific tasks (i.e., maximum performance). To more clearly differentiate socio-emotional skills from related constructs, Schoon proposes an integrative taxonomy of “domains and manifestations of social-emotional competences” (DOMASEC) that represent cross-cutting themes in research on social and emotional learning, personality, and motivation.

## Socio-Emotional Skills Predict Important Life Outcomes—Over and Above Cognitive Skills

Socio-emotional skills predict a broad range of important life outcomes, such as educational achievement (e.g., Poropat, 2009), income (e.g., Danner et al., 2020), reemployment success (e.g., Gnambs, 2017), health (e.g., Bogg and Roberts, 2004), and life satisfaction (e.g., Rammstedt et al., 2017)—often over and above cognitive skills as well as sociodemographic factors such as educational attainment (e.g., Spengler et al., 2015). Findings from Allen et al. for the years 2004–2017 suggest that certain types of socio-emotional skills may even have become more important in the labor market in recent decades.

Several contributions to the present issue provide additional evidence for the predictive power of socio-emotional skills for a broad range of outcomes in different life domains and life stages. The majority of these articles focus on academic achievement. They show that socio-emotional skills—measured with different frameworks—predict academic performance and flow experiences at school (Schmidt et al.; Steinmayr et al.; Wagner et al.) as well as successful transitions to the labor market (Nießen et al.)—over and above cognitive skills and socioeconomic status. Three other contributions address the relation between socio-emotional skills and job outcomes. Specifically, they demonstrate that socio-emotional skills predict adults' job performance over and above cognitive ability (Bergner; Harzer et al.) as well as participation in further training (Laible et al.).

As researchers or organizations, we have tended to focus on constructs that have demonstrated empirical associations with success or criterion variables in the past. However, focusing also on socio-emotional skills allows us to actively shape for the better environment in which we all, as individuals, learn, work, and live. For example, it has been found that less agreeable individuals tend to be more successful (e.g., Boudreau et al., 2001). Should universities or organizations therefore select more aggressive applicants as future students or employees? Perhaps not. Selecting applicants based on socio-emotional skills such as empathy, solidarity, honesty, or fairness may be a more promising approach.

## Socioemotional Skills Can Be Learned and Taught

Given the demonstrable importance of socio-emotional skills for success at school and at work, an essential question from a policy and practice perspective is whether these skills are malleable. There is broad agreement that—despite their substantial heritability—socio-emotional skills can be learned and shaped through education and interventions. However, it remains unclear how the development of these skills can best be fostered. Two articles in this issue contribute to this debate: Schiepe-Tiska et al. examine the role of teachers for social and emotional learning at school; Feron and Schils present evidence from a randomized controlled trial investigating whether self-reflection on school behavior can improve school performance. Their decidedly mixed findings illustrate a broader consensus in the field—namely, that researchers have yet to gain a complete understanding of how best to foster socio-emotional skills, for example, through curricular design or targeted interventions.

## Socio-Emotional Skills Can Be Assessed Economically—International Large-Scale Assessments Should Include them to Enable Further Research

The contributions in this Research Topic underscore that we do indeed need socio-emotional skills. In particular, the incremental predictive power of socio-emotional skills for engagement and success at school and at work is now abundantly clear. However, as discussed in detail in the articles in this Research Topic, there are several unresolved questions about socio-emotional skills that require more comprehensive data. Comprehensively assessing socio-emotional skills with instruments such as the Behavioral and Emotional Skills Inventory (BESSI; Soto et al., 2021) takes 15 min or less. This makes the assessment of socio-emotional skills as a complement to cognitive abilities attractive and cost effective. Without assessing socio-emotional skills also, researchers cannot achieve a complete understanding of individual differences in success, health, or social participation. Hence, we believe that more future studies—especially international large-scale assessments—in educational psychology, work and organizational psychology, and personality psychology should include measures of socio-emotional skills. Large-scale and ideally longitudinal data on socio-emotional skills will enable researchers to resolve long-standing questions, especially those regarding the development of these skills over the lifespan. It is now clear that socio-emotional skills can contribute to life success, broadly speaking. The most potent individual and contextual influences on the development of socio-emotional skills, and the most promising intervention strategies to foster their development, have yet to be identified. These formidable tasks for future research can build on the work in this Research Topic.

## Author Contributions

DD, CL, and MS wrote sections of the manuscript. All authors contributed to manuscript revision, read, and approved the submitted version.

## Conflict of Interest

The authors declare that the research was conducted in the absence of any commercial or financial relationships that could be construed as a potential conflict of interest.

## Publisher's Note

All claims expressed in this article are solely those of the authors and do not necessarily represent those of their affiliated organizations, or those of the publisher, the editors and the reviewers. Any product that may be evaluated in this article, or claim that may be made by its manufacturer, is not guaranteed or endorsed by the publisher.
